# Analysis of miRNA expression profiles in breast cancer using biclustering

**DOI:** 10.1186/1471-2105-16-S4-S7

**Published:** 2015-02-23

**Authors:** Antonino Fiannaca, Massimo La Rosa, Laura La Paglia, Riccardo Rizzo, Alfonso Urso

**Affiliations:** 1 ICAR-CNR, National Research Council of Italy, viale delle Scienze, Ed.11, 90145 Palermo, Italy; 2 ICAR-CNR, National Research Council of Italy, via P. Castellino 111, 80131 Napoli, Italy

**Keywords:** Next Generation Sequencing, miRNA expression profiles, breast cancer, biclustering

## Abstract

**Background:**

MicroRNAs (miRNAs) are important key regulators in multiple cellular functions, due to their a crucial role in different physiological processes. MiRNAs are differentially expressed in specific tissues, during specific cell status, or in different diseases as tumours. RNA sequencing (RNA-seq) is a Next Generation Sequencing (NGS) method for the analysis of differential gene expression. Using machine learning algorithms, it is possible to improve the functional significance interpretation of miRNA in the analysis and interpretation of data from RNA-seq. Furthermore, we tried to identify some patterns of deregulated miRNA in human breast cancer (BC), in order to give a contribution in the understanding of this type of cancer at the molecular level.

**Results:**

We adopted a biclustering approach, using the Iterative Signature Algorithm (ISA) algorithm, in order to evaluate miRNA deregulation in the context of miRNA abundance and tissue heterogeneity. These are important elements to identify miRNAs that would be useful as prognostic and diagnostic markers. Considering a real word breast cancer dataset, the evaluation of miRNA differential expressions in tumours versus healthy tissues evidenced 12 different miRNA clusters, associated to specific groups of patients. The identified miRNAs were deregulated in breast tumours compared to healthy controls. Our approach has shown the association between specific sub-class of tumour samples having the same immuno-histo-chemical and/or histological features. Biclusters have been validated by means of two online repositories, MetaMirClust database and UCSC Genome Browser, and using another biclustering algorithm.

**Conclusions:**

The obtained results with biclustering algorithm aimed first of all to give a contribute in the differential expression analysis in a cohort of BC patients and secondly to support the potential role that these non-coding RNA molecules could play in the clinical practice, in terms of prognosis, evolution of tumour and treatment response.

## Background

MicroRNAs (miRNAs), among small non coding RNAs (sncRNAs), are emerging as key regulators in multiple cellular functions, due to their crucial role in many important physiological processes (cell development, cell proliferation, apoptotic process, and response to different external and stress signals) [1]. Moreover it has been recently demonstrated that miRNAs are differentially expressed in specific tissues, during specific cell statuses, or in many diseases as tumours [2]. MiRNAs are small non coding molecules of single stranded RNA, 22-25 nt long, involved in the negative regulation of the gene expression to a post-transcriptional level. MiR-NAs in fact pair with specific RNA messengers (mRNA), causing their degradation or blocking the translational process of the protein product [3-5]. An interesting application involving these elements is the analysis of differential gene expression, obtained by comparing the transcriptional profiles of two or more individuals, tissues or cell types. Massive sequencing technique is an increasingly popular method used to study this mechanism. For instance, information on transcripts in healthy and diseased subjects may allow to identify which genes are expressed in a significantly different manner between these two groups and, thus, it is used to highlight differences that involve the pathological condition. Based on the evidence that groups of miRNAs are differentially expressed in several types of cancer, it is possible to study their involvement, negatively or positively, in the control of intracellular signalling pathways that have a direct influence on cancerous growth, such as proliferation and apoptosis. Therefore those miRNAs could act as oncogenes or tumour suppressors [3,6-11]. The importance of miRNAs in the regulation of gene expression is underlined also by the fact that few miRNAs can interact on many mRNAs. The altered expression of a single miRNA can lead to the deregulation of several molecular pathways, that may contribute separately, or cooperate, to the emergence of a malignant cell phenotype [1,12,13]. Many scientific works about differential gene expression analysis, show an association of miRNAs with development and tumour progression, as chronic lymphocytic leukemia (CLL), prostate cancer, non-small cell lung cancer (NSCLC), colorectal cancer (CRC) [2,7,14-17]. Volinia *et al *[18], reported a microarray analysis of miRNAs on 540 samples belonging to different tumour types (lung, breast, stomach, prostate, colon, pancreas), and showed a miRNA signature that join different solid tumours. Inside this signature were found miRNAs already known to be associated with other cancers, among them miR-17-5p, miR-20a, miR-21, miR-92, miR-106a, and miR-155. In this last decade more than 400 studies focusing on the role of miRNA expression levels and their regulation in breast cancer have been published [19]. Unfortunately, most of these studies were focused only on a specific subset of miRNAs, or a limited group of patients [20,21]. The role of microRNAs was also demonstrated in the early stages of the disease progression and metastasis. In fact, several experimental evidences showed the involvement of miRNAs in the regulation of biological processes that lead to the acquisition of metastatic potential, including adhesion, invasion, migration, epithelial-mesenchymal transition (EMT) and angiogenesis [22,23].

In the past decade, the microarray technique was successfully used for the analysis of gene expression but, due to some limitations [24-26], more recently microarray technology was accompanied by RNA sequencing (RNA-seq) method for the analysis of the transcripts. RNA-seq method belongs to Next Generation Sequencing (NGS) techniques and generates DNA sequences complementary to the RNA reference molecule. With respect to microarray, two main advantages of RNA-seq method are (1) the ability to quantify a large dynamic range of expression levels, with absolute rather than relative values, and (2) the absence of a priori knowledge of the sequence to be analysed [27].

Nevertheless, there is not a standard method used to analyse the gene expression by RNA-seq data. In order to assist biologists in the complex task of interpretation of the large amount of data produced by NGS techniques, an investigation technique should be based on computational methods.

Clustering methods have been usually used to analyse gene expression data, with the purpose to discover groups of genes (clusters) that are co-regulated with respect to certain experimental conditions. Traditional clustering approaches, like for instance k-means algorithm, however, have proved to perform well only with a small set of expression data. With large datasets, composed of thousands of genes and hundreds of experimental conditions, clustering techniques are not able to correct identify the gene clusters, because they consider the expression profiles under all conditions at the same time. Many regulation mechanisms, indeed, involve only a set of genes and a limited set of experimental conditions [28,29]. For this reason, in recent years biclustering algorithms have been developed for the study of gene expression data [30]. A bicluster is defined as a group of genes showing a similar regulation behaviour over a subset of experimental conditions. Moreover biclustering allows to obtain overlapping biclusters, in which a gene can be involved in different regulation patterns according to the groups of considered conditions. The biclustering approach can contribute to interpret the big amount of RNA-seq data in an easier, faster and specific way. Moreover, as reported by literature, miRNAs are deregulated in breast cancer as well as in other hematopoietic and solid tumours [2,6,14,19,20,23]. Clustering those molecules according to subclass of tumours by histological and molecular point of view can help to better define a potential role of miRNA as prognostic, diagnostic and therapeutic markers.

The use of biclustering algorithm in order to correlate miRNA profiles to specific tumour features was supported by a recent work [31]. It showed the use of a biclustering approach to detect breast cancer subgroups with common clinical features. The proposed approach can be a new useful method to analyse and stratify treatment and predict prognosis, with respect to not fixed sets of genomic biomarkers.

The present work, that is an extended version of [32], will take into account miRNA expression profiles in breast cancer samples and healthy controls, in order to identify some patterns of deregulated miRNA in human breast cancer and to give a contribution in the understanding of the mechanism involved in the regulation of this type of cancer.

## Methods

Biclustering is a computational procedure that separates homogeneous sub-matrices inside a matrix composed of objects vs features. In our study the data matrix is constituted by samples vs miRNAs. The biclustering procedure selects simultaneously a subset of features that characterizes a subset of samples, on the basis of a "local similarity" criterion. On the contrary, traditional clustering groups together objects that are similar with respect to the whole set of features, not to a subset of features [30]. It is also clear that a proper biclustering technique is also different from a two-steps clustering that acts first on rows and then on columns of the data matrix, such as the double hierarchical clustering implemented in [33]. Considering our study, biclustering will find a subset of miRNAs that are differentially expressed with regards to a subset of samples. Among the available biclustering tools, we consider the Iterative Signature Algorithm (ISA) [34].

**Figure 1:**
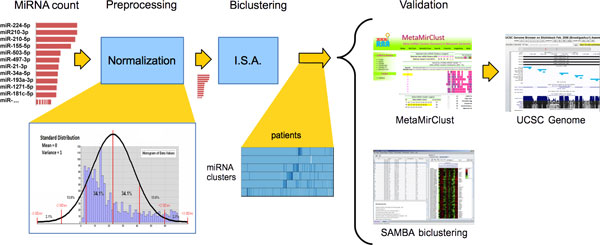
**The proposed pipeline**. Starting from miRNA count, we apply a normalization of data and perform a biclustering with ISA algorithm. The supervised evaluation exploits two online repositories, MetaMirClust and UCSC Genome browser, and validation by consensus with SAMBA algorithm.

Figure 1 shows the whole pipeline used in this work. The input is a matrix of miRNA expression profiles, preprocessed using a standard distribution normalization, as recommended by ISA's authors. Then ISA algorithm is executed and the resulting biclustering (miRNA clusters vs patients) is represented with a heatmap visualization. Finally, there is the three steps validation of each bicluster, using the MetaMirClust online repository [35], the analysis of literature collected in UCSC Genome browser [36] and the consensus of our results by means of another biclustering technique, the SAMBA algorithm [37].

### Iterative signature algorithm

According to recent surveys [38,39] that compare several biclustering algorithms on gene expression datasets, the ISA algorithm [34] is one of the most effective. Given a gene expression matrix, ISA algorithm discovers simultaneously sets of coregulated genes and the corresponding sets of experimental conditions. These biclusters are called *transcription modules *by ISA's authors. In our approach, the set of genes are represented by a set of miRNAs and the experimental conditions can be identified with breast cancer samples and healthy tissues.

Considering a gene expression matrix *E *of size *m *× *n *(with *m *= number of genes and *n *= number of samples), the ISA algorithm works in the following way. First of all, it is created a random sparse 0/1 vector of size *m*, called seed vector *r*_0_. The elements of *r*_0 _are used to select a subset of the genes (rows). *E *is then transposed (*E'*), it is multiplied by *r*_0_, and the result is thresholded, obtaining as result the vector *c*_0 _of size *n*. This threshold is obtained computing the mean and standard deviations of the vector *c*_0_, and considering only the elements that have a standard deviation from the mean higher than the threshold. The vector *c*_0 _is then used to select a subset of the input samples, considering only the columns of *E *corresponding to the non-zero components of *c*_0_. After that, *E *is then multiplied by *c*_0_, and the result is again thresholded in order to obtain the vector *r*_1 _(of size *m*). This procedure is iteratively repeated until either *r*_*i*-1 _and *r_i_*, and *c*_*i*-1 _and *ci *are close enough, according to a convergence criteria defined in terms of Pearson correlation. The final vectors *r_final _*and *c_final _*are used to extract a subset of the genes and samples, composing the biclusters, from the expression matrix *E*. Genes are selected considering the rows of *E *corresponding to the the non-zero components of *r_final_*; samples are selected considering the columns of *E *corresponding to the the non-zero components of *c_final_*. Therefore the two most important parameters of ISA algorithm are the two thresholds: *th_r _*for the matrix rows (representing the genes), *th_c _*for the matrix columns (associated to the samples). The threshold values affect the composition of the biclusters: for example, if the row (gene) threshold is high, then the biclusters will have very similar genes. Lower threshold values, in turn, will provide bigger biclusters with less similar genes. The same behaviour applies when considering the column (sample) threshold. Moreover the biclusters are "soft", meaning that they allow overlapping among genes and/or samples, and, according to the threshold values, it is possible that groups of genes and samples do not belong to any bicluster. It is also possible to obtain a bicluster that correlates a single gene with a group of patients and vice versa. As the authors state, ISA algorithm has the best performance processing normalized input data, i.e the input matrix have zero mean and standard deviation equal to one. In addition to the biclusters, ISA algorithm also provides as output two numeric matrices: one for the row (gene) components of the bicluster, and another one for the column (sample) components of the bicluster. Considering for example the resulting matrix for the genes, each column corresponds to a bicluster. The rows with a non-zero value belong to the bicluster related to that column. These matrices have values ranging from -1 and +1 and they express the association degree of a gene, or sample, to that bicluster. If two genes belong to the same bicluster with the same sign, they are correlated, otherwise they are anti-correlated. In the following discussions, for sake of simplicity, we take into account the output matrix having samples as rows, and the miRNA biclusters as column. Since the biclusters are unique, the same conclusions hold when considering miRNA vs sample biclusters.

In our work, we used the ISA implementation provided by the R package *isa2 *[40].

### Validation technique

We adopted two different validation criteria: 1) semi-supervised evaluation criterion, based on scientific literature; 2) consensus with another biclustering tool, the SAMBA algorithm [37], that has proven to discover relevant subsets of genes and samples from gene expression data [41]. Indeed, since we cannot perform *in vitro *experiments, we referred to: 1) an external repositories providing informations about different groups of miRNAs in human species; 2) a different biclustering algorithm to compare our results with.

In particular, we investigated over MetaMirClust database [35] and UCSC Genome browser [36]. The former is a repository of miRNAs in animalia kingdom that provides a useful bioinformatics resource to facilitate the advanced interrogations on the composition of miRNA clusters. The latter is a genome browser containing a collection of annotation for several genomic regions and, for each annotation, it reports its description, details about its construction, methods used in the experimentation, validation, credits, and references. Exploiting these two services, we could compare miRNA clusters obtained by the proposed technique with those contained in MetaMirClust and then we found for each bicluster all available literature references in UCSC Genome browser. Finally, we got information from references, in order to examine how they are related to differential expression of miRNAs in breast cancer. Our biclustering results were further validated using the SAMBA biclustering algorithm by checking if it is able to discover the same biclusters obtained through the ISA algorithm.

## Results and discussion

In this section, we introduce the experimental dataset and then we discuss the biclustering results obtained according to the proposed pipeline, introduced in the Section Methods.

### Experimental dataset

We used a breast cancer dataset composed of a large cohort of patients. RNA-seq data were collected by Farazi *et al *([33]) and uploaded in Sequence Reads Archive data bank (SRA, http://www.ncbi.nlm.nih.gov/Traces/sra/sra.cgi). The miRNA expression profiles were downloaded from Gene Expression Omnibus (GEO) with accession number *GSE28884*. The dataset is composed of 169 tumour samples, 10 healthy tissues, 6 cell lines (MCF7, MCF10A, HCC38, BT474, MDA-MB134, and ZR-751), for a total of 185 samples. We wanted, indeed, to test the efficacy of the ISA biclustering algorithm on a real dataset, not altered by a guided choice of the samples, and on a real life problem as the breast cancer analysis. Another parameter that we took into account was the partition of samples according to the clinical and histo-pathological features, molecular sub-type and the presence of one or more immune-histo-chemical (IHC) markers (ER, PR, Her2 status). Our aim is in fact to discover relationships between specific patterns of patients and specific groups of miRNA.

Our biclustering approach allowed us to evaluate miRNA deregulation in the context of miRNA abundance, and tissue heterogeneity, important elements in identifying miRNAs that would be useful prognostic and diagnostic markers.

**Figure 2:**
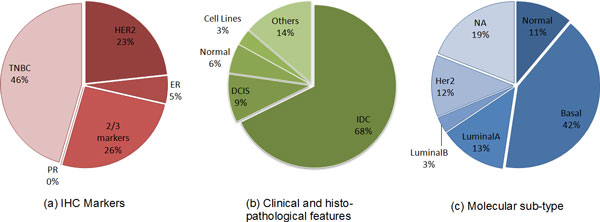
**Dataset Composition**. The real world dataset is composed by 185 samples. Each pie represents a different characteristic, where the percentage values give information about the composition of the dataset.

Figure 2 shows the dataset composition. In order to verify if there could be some potential link between miRNA biclusters and some specific tumour characteristic, we considered three features (the list of patient features was provided by Farazi *et al*. [33]): a) presence or absence of IHC markers; b) clinical and histo-pathological features; c) molecular sub-type. For the IHC markers, point (a), it was considered the presence or absence in a tumour sample of Estrogen Receptor (ER), Progesteron Receptor (PR), or the ERB2/Her2 protein. As it can be seen the majority of patients are triple negative (46%). From a molecular point of view these patients could be relevant to deeper understand the biology of these tumours and potentially to give answers in terms of pharmacological treatments. Regarding the clinical and histopathological features, point (b), patients are divided in IDC (Invasive Ductal) (68%), DCIS (Ductal carcinoma In Situ) (9%), Normal samples (healthy controls) (6%), Metaplastic (5%), Cell lines (3%) and others (atypical medullary, apocrine, adenoid, mucinous A). It can be noticed that also this feature is non-homogeneous as the most part of analysed samples are IDC, compared to all the other tumour samples and healthy controls. The third characteristic, point (c) that we considered was the molecular sub-type, shared in basal (42%), Luminal A (13%), Her2 (12%), Normal (11%), Luminal B (3%) and 19% of remaining samples were NA, because we did not have information regarding their molecular sub-type.

Regarding the miRNA profiles, we set the threshold for sample comparison to 5,000 total miRNA sequence reads per replicate merged library, as reported in [33], identifying 231 miRNA. miRNA profiles of a sample have been presented as relative percent (%) miRNA read frequencies (rf) by dividing miRNA read counts by total miRNA reads per library. Furthermore, miRNA profiles have been condensed by assigning individual miRNA to sequence families (denoted by *sf-miR*). This approach reduces the complexity of the data. The sequence families are most informative for characterization of seed-sequence-dependent miRNA-target regulation. We considered the miRNA families as identified in the work of Farazi *et al*. [33] and provided in the Supplementary Table S4 available from the supplementary material of their work.

### Experimental results

**Figure 3:**
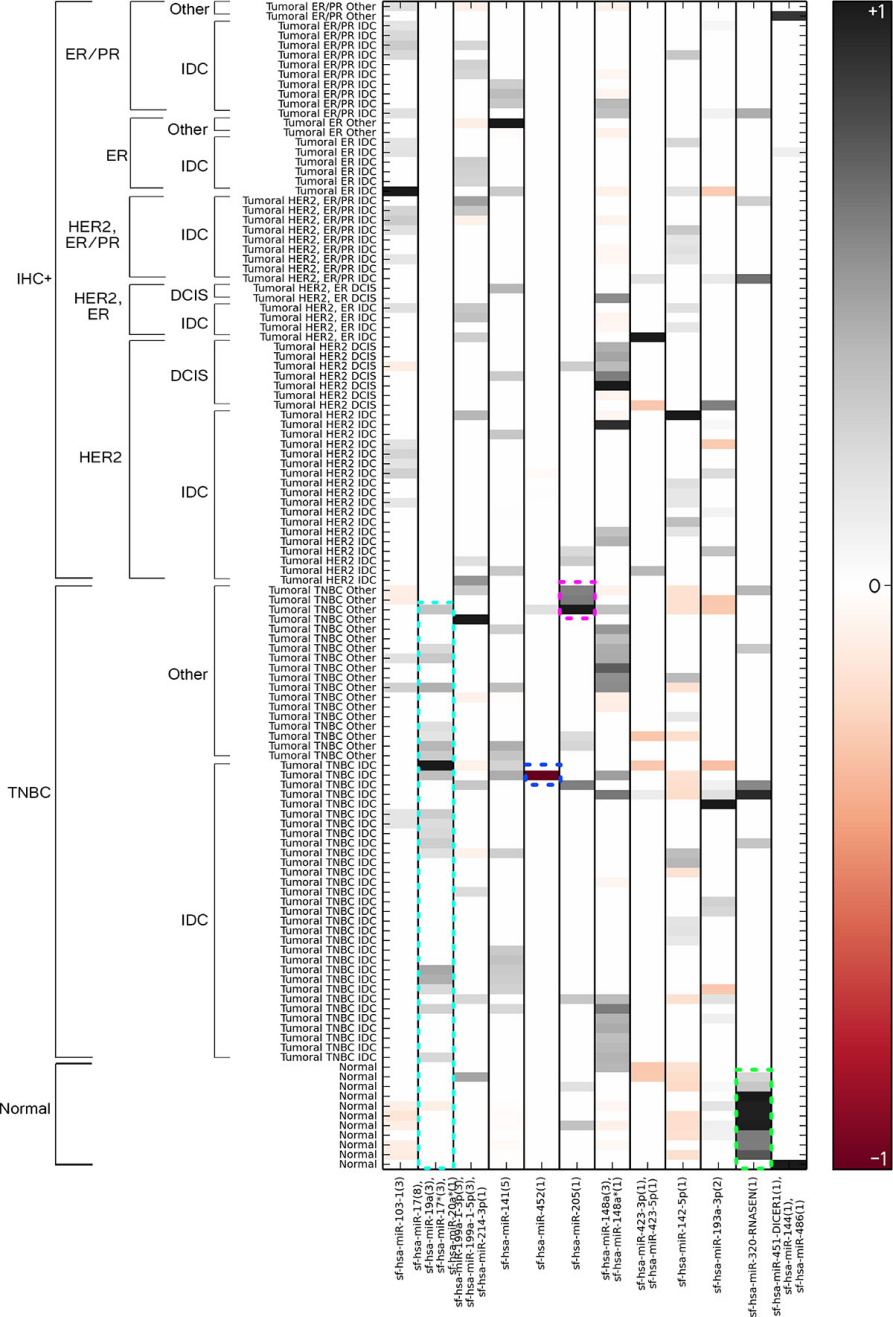
**Bicluster visualization**. The heatmap shows the membership of each patient with regards to the 12 miRNA biclusters. Only the patients belonging to at least one miRNA bicluster are reported. Membership ranges from -1 to +1, with 0 meaning no membership. +1 and -1 indicate highest membership, but with opposite correlation degree. The heatmap indicates a strong relationship between some features of the dataset and specific subclass of miRNA. Indeed the expression patterns of miRNA family hsa-mir-17/19a/17*/20a* inside the azure rectangle shows an almost exclusive link with TNBC samples. The pink rectangle in miRNA-205 links this non coding RNA to a specific subclass of BC samples. The blue rectangle evidences an inverse correlation between the mir-452 and some IDC samples. The grey rectangle evidences the association to mir-320-RNASEN(1) with histological normal samples

**Figure 4:**
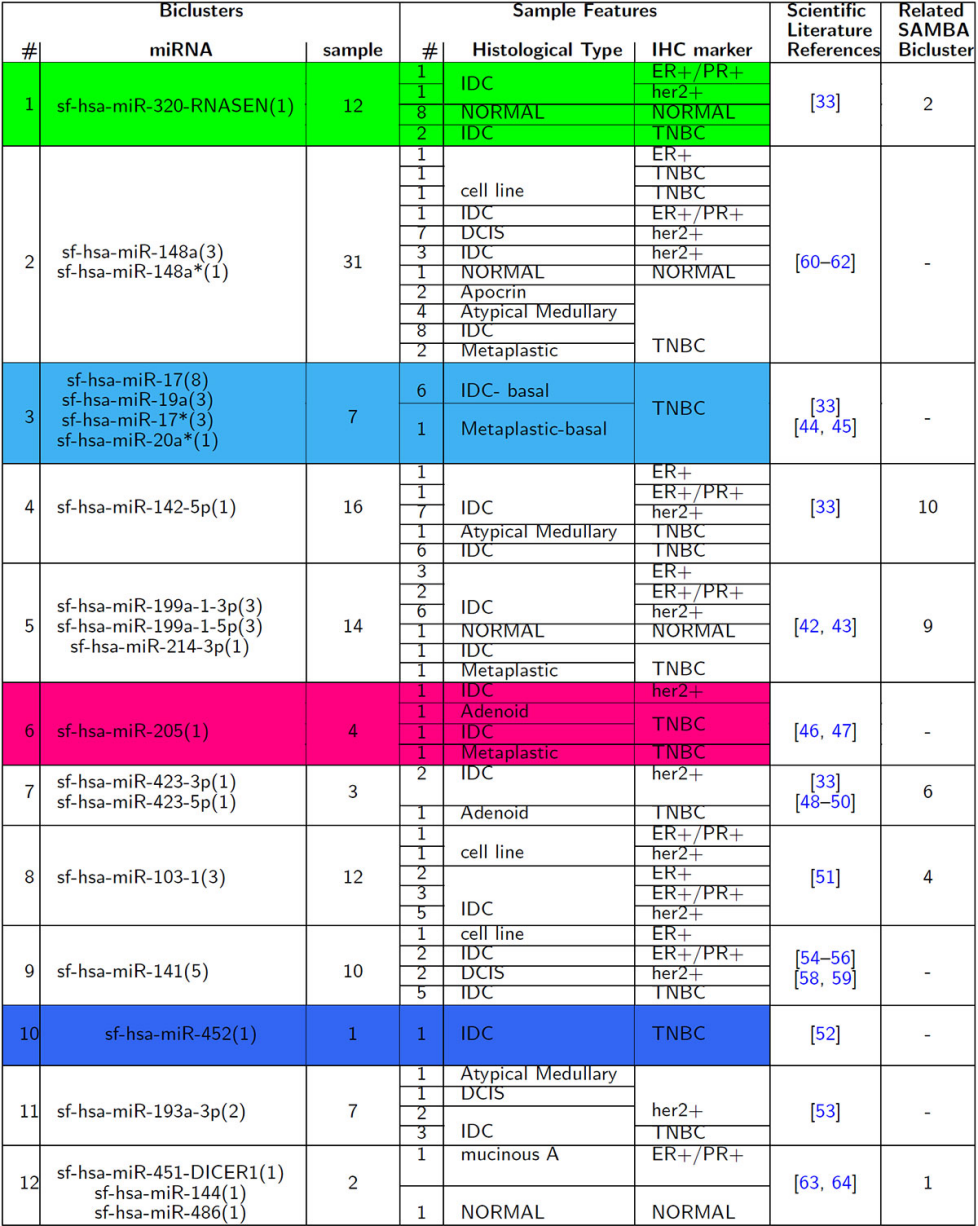
**Table of biclustering results**. It reports, for each obtained bicluster, the group of miRNAs and the number of patients. In addition, for each patient in bicluster, some main characteristics are reported. The last two columns contain references provided by literature works and the number of the related bicluster, if any, discovered by the SAMBA algorithm. All the biclusters obtained with SAMBA algorithm are provided in the Supplementary material, Additional file 1 and 2. See caption of Figure 3 for details about red, blue, azure and green rectangles.

We aimed to evaluate the differential expression of miRNAs in breast cancer samples versus healthy tissues. Figure 3 shows the results obtained by the ISA algorithm. Membership values, ranging from -1 to +1, are showed for every patient and each miRNA bicluster, as produced by the ISA algorithm. Starting from the heatmap visualisation, we considered the patients belonging to the miRNA bicluster with the highest membership value. The differential expression analysis evidenced 12 different miRNA clusters. Each of these was associated to a specific cluster of patients. Figure 4 summarizes the composition of each bicluster, their references in scientific literature and if they were also discovered by the SAMBA algorithm, as explained in Section *Validation Technique*. Since the analysis of all the biclustering results obtained through SAMBA algorithm is not in the scope of this work, we provide them in the Supplementary Material, Additional file 1 and 2, for sake of completeness.

The two thresholds of the ISA algorithm were regulated in order to have a manageable (not too large) and meaningful number of biclusters. *th_r _*was set equal to 3.5, *th_c _*was set equal to 1.0. Other threshold values gave few clusters but with many elements, or many clusters with few elements. Considering the random initialization of ISA algorithm we ran it ten times with different initialization values but with the same thresholds, in order to assess the stability of the 12 biclusters. Each time we obtained the same 12 biclusters, although with a different sorting. As it is evident in Figure 4, 7 out of 12 biclusters are composed of one sequence family. Unlikely traditional clustering methods, where a cluster with only one element has often very low importance, a bicluster that correlates a single element with a group of features give us a lot of information.

As it will be discussed in the rest of this section, indeed, biclusters composed of one single miRNA family linked to a group of samples, enable us to associate a particular miRNA family to a set of sample tissues with well defined immuno-histochemical and histological features. This characteristic of the algorithm allows in a "one step" process to obtain results that are normally produced with more passages with a classical hierarchical clustering process.

Our biclustering procedure highlighted many results from the original work of Farazi *et al *[33], it also produced other results that are confirmed by other works in the literature, and some new results that need more investigation. First of all we comment the results in accordance with the original Farazi work.

The first step of our analysis was to check if the healthy tissues were univocally associated to the same sub-class of miRNA. The data showed an exclusive association between these samples and the miRNA cluster sf-hsa-miR-320-RNASEN (green rectangle on Figure 3 and Figure 4). The obtained data were confirmed by Farazi *et al*. [33] and by the SAMBA algorithm (bicluster 2, available in the supplementary material, Additional file 1). Moreover, as it will be argued, the algorithm showed the association between specific sub-class of tumour samples having the same immuno-histo-chemical (IHC) and/or histological features and miRNA clusters, as can be evidenced for "atypical medullary" and DCIS samples that are linked to sf-hsa-miR-148a* and sf-hsa-miR-148a clusters. The bicluster evidenced also an over-expression of sf-hsa-miR-142-5p in Her2+/IDC samples compared to other classes of tumour samples, as DCIS or TNBC, and compared to healthy controls. These results were in accordance with the evidence reported by Farazi *et al *[33]. SAMBA biclustering showed an association between this miRNA bicluster, ER+/PR+/IDC, and HER2+/IDC samples, confirming our results. Indeed there is an over-expression of this group of microRNA with regards to these classes of samples. Moreover the algorithm reported a down-expression of TNBC samples for this miRNA cluster (supplementary material, Additional file 1 bicluster 10). Some IDC samples have shown an association to miRNA sf-hsa-miR-199a-1-3p, sf-hsa-miR199a-1-5p e sf-hsa-miR-214-3p clusters, that were over-expressed. Schwarzenbach *et al*. and Shatseva *et al*. confirmed our data clustering, showing increased levels of mir-214 in serum of BC patients compared to healthy controls [42,43]. The analysis with SAMBA algorithm reported also an over-expression for ER+/PR+/IDC, Her2+/IDC samples, confirming our results; it also reported a down-expression for some TNBC samples not evidenced with ISA biclustering (supplementary material, Additional file 1 bicluster 9).

Triple Negative Breast Cancer samples (TNBC) are a distinct group of tumour samples characterized from distinct histological and IHC features, as they lack for the presence of estrogen receptor (ER), progesterone receptor (PR), and Her2 protein receptors. They are associated to a specific pattern of miRNA, that is disregulated: sf-hsa-miR-17, sf-hsa-miR-19a, sf-hsa-miR-17*, and sf-hsa-miR-20a* (azure rectangle on Figure 3 and Figure 4). These are oncogenes and their targets are "tumour suppressors" of the cell cycle. Many scientific works report an over-expression of this cluster of miRNA in different types of solid tumours and hematopoietic diseases [33,44]. Especially, this association involves a specific sub-class of TNBC that is the "basal like subtype", as reported also by De Rinaldis *et al*. [45]. This evidence suggests the tendency of specific miRNA that are "sub-type specific" to influence the expression levels of their targets. The identification of a cluster of miRNA specifically linked to the TNBC sub-class of samples is very important because nowadays there are not targeted therapies for this BC sub-class. The discovery of miRNAs associated with TNBC could mean to find a less invasive strategy of intervention. Differently from our results, SAMBA biclustering reported a down-expression for miR-17* and miR-20* in TNBC samples (supplementary material, Additional file 1 bicluster 7).

In literature, data about the role of miRNA-205 are conflicting: our biclustering approach highlighted an over-expression of this miRNA in some IDC, adenoid and metaplastic samples (pink rectangle on Figure 3 and Figure 4). Interestingly, a closer look to the IHC features evidenced that this specific class of miRNA strongly associates to TNBC samples, as the values reported by the algorithm are almost equal to 1 in absolute value. This data was in accordance with the result obtained by Farazi *et al*. [33]. No association was found for this bicluster with SAMBA analysis. Other miRNA profiling has shown increased levels of this miRNA-205 in BC samples without vascular invasion compared to those with vascular invasion. Moreover miR-205 has been shown to be down-regulated in BC tumour samples and BC cellular lines [46,47].

miR-423-3p and miR-423-5p are the mature transcripts of miR-423. Many scientific works reported that their expression was altered in different forms of cancer type [33,48-50]. Farazi *et al*. reported that these two the mature forms of miR-423 were highly expressed in ductal infiltrating BC that subsequently give metastasis, underlying the prognostic independent role of the disease [33]. Our biclustering analysis evidenced a distinct group of IDC patients where miR-423 was over-expressed. SAMBA biclustering showed an association with only miR-423-5p and some distinct groups of samples: an over-expression was associated to Her2+/IDC samples, and a down-expression was found with ER+/PR+/mucinous A samples (supplementary material, Additional file 1 bicluster 6).

The proposed biclustering procedure gave some new results discussed below; many of these findings are confirmed by other papers in the literature. As general observation we found that most of the results are related to over-expressed miRNAs, with only one bicluster (row 10 in Figure 4) involving down-regulated miRNAs.

The differential expression analysis of sf-hsa-miR-103-1 showed an over-expression in IDC cluster samples, positive for at least one of three IHC marker (PR/ER/Her2). These results have been confirmed by SAMBA biclustering (supplementary material, Additional file 1 bicluster 4). A recent work of Wang X. evidenced a potential role of this microRNA as serum biomarker in BC patients. Moreover its expression correlates with an advanced state of the disease (P < 0.05) and with linfonodal metastasis (P < 0.05) [51].

The sf-hsa-miR-452 is involved in different processes as cell-cell interaction, cell growth and proliferation, inflammatory response and apoptotic processes. The biclustering algorithm showed a strong down-expression of this miRNA family (blue rectangle on Figure 3 and Figure 4). Interestingly Van Schooneveld *et al*. [52] referred an over-expression in healthy patients compared to BC patients, and they evidenced a positive association (P < 0.05) between expression levels of miR-452 in serum of patients and the number of methylated genes in plasma. The experimental evidences showed a strong over-expression of miR-452 in healthy controls compared to tumour samples, and because of the evidence of the stability of this miRNA in plasma, the authors speculated a potential role as biomarker for the detection of BC in human plasma. SAMBA biclustering reported no association for this miRNA.

The biclustering analysis showed an over-expression of sf-hsa-miR-193a-3p in IDC sub-class of patients. An over-expression of this miRNA in tumour samples was previously evidenced in other works as reported by Fung Lin Yong [53]. No association was found with SAMBA algorithm.

Our algorithm produced also some results that are often discordant from literature: miR-141 and miR-200c belong to a miRNA family of important regulator of cellular transition from epithelial to mesenchymal (EMT) [54-56]. Moreover, miR-200c has been shown to play a role in disregulation of normal expression patterns of different cancer types [18,57]. Our analysis evidenced an over-expression of sf-hsa-miR-141 in tumour samples compared to healthy controls. Most of them are IDC, and Her2 negatives. In literature data are contrasting [58,59]. Moreover SAMBA biclustering reported no results for this miRNA bicluster.

As previously said, sf-hsa-miR-148a* and sf-hsa-miR-148a miRNA clusters were linked by the algorithm analysis to "atypical medullary" and DCIS samples. Also these data differ from literature: scientific works conducted on different cancer types showed a down-regulation in tumour samples compared with healthy controls [60-62]. No association was found with SAMBA biclustering.

A new association is reported about the miRNA bicluster composed of sf-hsa-miR-451-DICER1, sf-hsa-miR-144, sf-hsa-miR-486: our biclustering showed an over-expression of this bicluster in a sub-class of IDC/her2 positive patients. In literature there are no data regarding an association about this last miRNA bicluster and breast cancer but there are more evidence about an over-expression of miRNA 451 in BC and other tumour samples [63,64]. Instead in [33] it is reported that miR-144 and miR-451 clustered together but were abundant in normal breast and reduced in IDC. Interestingly SAMBA algorithm also reported the association among miR-451-DICER1, miR-144 and miR-486. For this miRNA bicluster was evidenced an over-expression in ER+/PR+ samples (supplementary material, Additional file 1 cluster 1).

## Conclusions

In this paper we used a biclustering approach, by means of the ISA algorithm, in order to analyse the differential expression of miRNA in breast cancer samples. Thanks to its features, the biclustering method allows to cluster subset of patients with subset of miRNAs, exploiting this way miRNA biclusters under different samples conditions. Our analysis highlighted 12 miRNA biclusters, each of them involving different types of tumour samples, characterized by immuno-histo-chemical, histopathological and molecular sub-type features. Biclusters were validated in the current scientific literature with the support of the MetaMirClust and UCSC Genome Browser online tools. Further validation was provided by the consensus with another biclustering tool, the SAMBA algorithm. The proposed biclustering methodology has proved a valid instrument for the study of miRNA expression profiles, with the possibility to identify biclusters that can provide novel relationships among groups of miRNAs and patient conditions. Of course, discordant or new results need to be deeply investigated.

## Competing interests

The authors declare that they have no competing interests.

## Authors' contributions

AF: project conception, implementation, discussions, assessment, writing. MLR: project conception, implementation, discussions, assessment, writing. LLP: project conception, experimental tests, discussions, assessment, writing. RR: project conception, discussions, assessment, writing. AU: project conception, discussions, assessment, writing, funding. All authors read and approved the final manuscript.

## Supplementary Material

Additional file 1 **SAMBA biclustering results**. Excel file summarizing the whole biclustering results obtained with SAMBA algorithm. Each sheet of the file reports the composition, in terms of miRNA and sample, of each bicluster. Click here for file

Additional file 2 **Bicluster heathmaps**. An archive containing the heatmaps of each bicluster obtained with SAMBA algorithm. Click here for file

## Declarations

The publication costs for this article were funded by the project PO-FESR SICILIA 4.1.1.1 - "MIRCO - MicroRna in Clinica Oncologica".

This article has been published as part of *BMC Bioinformatics *Volume 16 Supplement 4, 2015: Selected articles from the 9th IAPR conference on Pattern Recognition in Bioinformatics. The full contents of the supplement are available online at http://www.biomedcentral.com/bmcbioinformatics/supplements/16/S4.
